# Role of Vitamin D Receptor (BsmI-VDR) and Insulin Receptor (NsiI-A/G) Gene Polymorphisms in Colorectal Adenoma Susceptibility

**DOI:** 10.3390/ijms25168965

**Published:** 2024-08-17

**Authors:** George Ciulei, Olga Hilda Orășan, Angela Cozma, Vasile Negrean, Ioana Para, Lorena Ciumărnean, Nicoleta Leach, Roxana Liana Lucaciu, Adriana Corina Hangan, Lucia Maria Procopciuc

**Affiliations:** 1 4th Department of Internal Medicine, Faculty of Medicine, “Iuliu Hațieganu” University of Medicine and Pharmacy, 400012 Cluj-Napoca, Romania; george.ciulei@umfcluj.ro (G.C.); angelacozma@yahoo.com (A.C.); vasile.negrean@umfcluj.com (V.N.); ioana.para@yahoo.com (I.P.); lorena_ciumarnean@yahoo.com (L.C.); nicoleta_leach@yahoo.com (N.L.); 2 Department of Pharmaceutical Biochemistry and Clinical Laboratory, Faculty of Pharmacy, “Iuliu-Hațieganu” University of Medicine and Pharmacy, 400012 Cluj-Napoca, Romania; liana.lucaciu@umfcluj.ro; 3 Department of Inorganic Chemistry, Faculty of Pharmacy, “Iuliu-Hațieganu” University of Medicine and Pharmacy, 400012 Cluj-Napoca, Romania; adriana.hangan@umfcluj.ro; 4 Department of Medical Biochemistry, Faculty of Medicine, “Iuliu Hațieganu” University of Medicine and Pharmacy, 400012 Cluj-Napoca, Romania; l.procopciuc@umfcluj.ro

**Keywords:** colorectal adenoma, vitamin D receptor, insulin receptor, gene polymorphisms

## Abstract

Vitamin D deficiency and type 2 diabetes mellitus are risk factors for colorectal cancer, suggesting a role for vitamin D receptor (VDR) and insulin receptor (INSR) gene polymorphisms. We investigated the prevalence of the VDR-BsmI (rs1544410) and NsiI A/G-INSR (rs2059806) polymorphisms and their associations with colorectal adenoma (CRA) in a Romanian population. A case–control study was conducted with 110 participants (67 with CRA and 43 controls) who underwent colonoscopy. Polymerase chain reaction–restriction fragment length polymorphism analysis was used to determine the genotype and allele frequencies of the two polymorphisms. Regarding rs1544410 and CRA patients, genotype distribution was 35% B/B, 47% B/b, and 19% b/b. In the controls, the distribution was 21% B/B, 45% B/b, and 34% b/b. For rs2059806, 12% of CRA patients had A/A, 30% A/G, and 58% G/G, while 8% of the controls had A/A, 40% A/G, and 52% G/G. The recessive model showed an odds ratio of 2.84 (95% CI: 1.04–7.72, *p* = 0.033) for the b/b genotype. CRA patients with b/b or G/G genotypes were diagnosed at a younger age. The b allele of the rs1544410 was a risk factor for CRA. Patients with the b/b and G/G genotypes were diagnosed earlier.

## 1. Introduction

Colorectal cancer (CRC) remains the third most diagnosed cancer (10% of global cancer incidence) and the second leading cause of cancer death worldwide (9.4%). The incidence of CRC in Romania in 2020 was 34.6/0000, representing 12.7% of all cancers diagnosed. The increase in CRC cases is primarily linked to increased exposure to environmental risk factors, driven by changes in lifestyle and the westernization of diet patterns [1,2]. Epidemiological studies have shown a link between vitamin D deficiency and an increased incidence of CRC [3,4]. Vitamin D insufficiency is a widespread issue affecting more than a billion individuals globally. This condition disproportionately impacts pregnant women, individuals with darker skin tones, or those who are obese. The primary reason for this deficiency is inadequate exposure to sunlight, with skin pigmentation and aging being significant physiological factors that reduce vitamin D synthesis [5]. Once produced in the skin or ingested, vitamin D3 undergoes a transformation in the liver, catalyzed by the enzymes CYP2R1 and CYP27A1, to become 25(OH)D_3_. This is then further processed in the kidneys into the active form, 1,25(OH)_2_D_3_ (calcitriol), through the action of the enzyme CYP27B1. This enzyme is regulated by parathormone and fibroblast growth factor 23 [6].

The active form of vitamin D may play a role in preventing cancer development through its antimitotic, pro-differentiation, and pro-proliferative effects. Calcitriol influences the expression of specific genes by interacting with the vitamin D receptor (VDR). In the early stages of colorectal cancer (CRC), high levels of CYP27B1 and VDR are found in well-differentiated tumors, with levels decreasing in less differentiated tumors [7]. Over 60 single nucleotide polymorphisms (SNPs) have been identified in the VDR gene. Polymorphisms, such as BsmI, ApaI, Tru9I (located in intron 8), and TaqI (in exon 9), are located near the 3′ untranslated region of the gene. These polymorphisms do not alter the amino acid sequence but are closely linked and form a haplotype block that influences mRNA stability and the transcription of the gene. The mutant b allele of the *VDR-BsmI* polymorphism (rs1544410) has been shown to be significantly correlated with CRC risk in several studies [8,9]. 

Clinical research indicates that individuals with elevated visceral fat and high blood glucose levels and who are overweight or obese face an increased risk of CRC [10]. Furthermore, type 2 diabetes mellitus (T2DM) has been identified as a significant risk factor for developing CRC [11]. The biological connections between T2DM and CRC include increased insulin and IGF axis activity, elevated blood sugar levels, inflammation caused by adipose tissue dysfunction, gastrointestinal motility issues, and weakened immune surveillance. The mitogenic and antiapoptotic properties of IGF-1 indicate that it is a growth factor involved in the onset and progression of CRC. IGFs exert their effects on cells through their specific attachment to several types of membrane receptors: type I (IGF-1R), type II (IGF-2R), insulin receptor (INSR), and a hybrid receptor (IGF-1R/INSR) [8]. Patients with T2DM also have an increased risk of colorectal adenoma (CRA) [12]. The rs2059806 SNP, a G-A variation in exon 8 of the INSR gene (*NsiI A/G-INSR*), has been observed to be associated with an increased risk of T2DM [13,14]. 

Given their association with CRC pathogenesis, the aim of our study was to determine the prevalence and significance of the *VDR-BsmI* and *NsiI A/G-INSR* polymorphisms in a Romanian population group diagnosed with CRA.

## 2. Results

The demographics and clinical characteristics of the patients and controls, stratified by sex, are summarized in Table 1. The male patients had a mean age of 66.8 ± 9.7 years, which was significantly greater than that of the control group (61.0 ± 10.4 years; *p* = 0.03). Overall, the patients included in the case group were older than the controls (*p* = 0.006). No significant differences were observed in the BMI, abdominal circumference, or smoking history between the patients and controls. There was no significant difference in the number of multiple adenomas, polyps with high-grade dysplasia, traditional serrated or sessile serrated polyps, or polyps larger than 1 cm between women and men. The distribution of polyps in the right colon was also not significantly different.

### 2.1. VDR-BsmI and NsiI A/G-INSR Polymorphism Distribution

Table 2 compares the allelic and genotypic distributions of SNPs in the *VDR-BsmI* and *NsiI A/G-INSR* genes between CRA patients and control subjects. The analysis was conducted using various genetic models, including codominant, dominant, recessive, and overdominant models.

According to the codominant model, individuals with the b/b genotype exhibited an increased risk for CRA, with ORs of 3.08 (95% CI: 1.04–9.12) unadjusted and 3.43 (95% CI: 1.07–11.01) adjusted for age, sex, and smoking status, indicating a statistically significant association. The B/b genotype showed increased ORs (1.61 unadjusted, 95% CI: 0.64–4.04; 1.37 adjusted, 95% CI: 0.50–3.75), although these differences were not statistically significant. 

The dominant model, which combined B/b and b/b against the reference B/B, yielded ORs of 2.03 (95% CI: 0.86–4.79) for the unadjusted model and 1.94 (95% CI: 0.77–4.88) for the adjusted model, both of which lacked statistical significance. In the recessive model, where b/b was compared against B/B + B/b, the adjusted model showed a significant OR of 2.84 (95% CI: 1.04–7.72, *p* = 0.033) with a Q value < 0.10. The overdominant model did not demonstrate any significant associations.

The codominant model analysis showed that neither the “A/G” nor the “G/G” genotype was significantly associated with CRA risk, with wide confidence intervals (OR = 2.08, 95% CI: 0.51–8.47 for A/G; OR = 1.40, 95% CI: 0.37–5.36 for G/G). The dominant model (A/G + G/G vs. A/A) and the overdominant model also failed to demonstrate significant associations. According to the recessive model, when G/G was compared with A/A + A/G, there was no increase in the risk of CRA (OR = 0.79, 95% CI: 0.36–1.71 unadjusted; OR = 0.91, 95% CI: 0.39–2.08 adjusted).

### 2.2. Comparison of VDR-BsmI and INSR Genotypes With Clinical Outcomes 

Table 3 shows the results of clinical comparisons between patients with CRA and controls in terms of the *VDR-BsmI* genotype and the *NsiI A/G-INSR* genotype. The focus was on the differences between the major and minor allele carriers, with comparisons between subjects with either the B/B or B/b genotype vs. b/b in the case of VDR-BsmI and A/G or A/A vs. G/G in the case of NsiI A/G-INSR. In the general population of patients and controls, no significant age difference was noted between the B/B + B/b (64.63 ± 10.93 years) and b/b (61.29 ± 8.97 years) genotypes (*p* = 0.13). However, within the subgroup of patients with CRA only, a significant difference in age was observed, where subjects with the b/b genotype were younger (62.39 ± 8.59 years) than those with BB + Bb (67.66 ± 10.72 years; *p* = 0.04). Similarly, for the *NsiI A/G-INSR* polymorphism, the entire cohort showed no significant difference in age between the A/A + A/G (65.66 ± 9.16 years) and G/G (62.05 ± 11.25 years) genotypes (*p* = 0.07). However, in the CRA-only group, a significant difference in age was observed, with the G/G genotype carriers being younger (62.83 ± 11.62 years) than the A/A + A/G genotype carriers (69.16 ± 7.46 years; *p* = 0.01). No other significant differences were noted for BMI, abdominal circumference, or traits such as smoking status, family history of CRC, or histological type of adenoma.

## 3. Discussion

Several authors have studied the *BsmI* polymorphism and its relationship with CRC risk. In one systematic meta-analysis, the B genotype was related to a decreased risk of CRC (B/B vs b/b, OR = 0.87, 95% CI: 0.8–0.94, *p* < 0.001) [15]. Another meta-analysis showed that the presence of the B allele was protective against CRC (B vs. b: OR = 0.86, 95% CI: 0.76–0.97, *p* < 0.001) [16]. The *BsmI* polymorphism does not affect the concentrations of 25(OH)_2_D_3_ or 1,25(OH)_2_D_3_ [17]. The *BsmI* site is in strong linkage disequilibrium with other polymorphisms within the VDR gene, such as the ApaI, TaqI, and poly(A) microsatellites. *BsmI* may play a role in VDR transcription, translation, or RNA processing. Individuals with the *BsmI* allele exhibit significantly elevated levels of erbB-2 expression, indicating the involvement of other tumor-related molecules in the functional effects of the *BsmI* polymorphism [15]. Regarding colon polyps, a 2011 meta-analysis of five studies revealed no association between the *BsmI* polymorphism and CRA [18]. In one later study, the presence of the ancestral B allele significantly reduced the risk for CRA in females (OR = 0.23, 95% CI: 0.09–0.63, *p* = 0.005) [19]. In our study, a significant association was observed between the b/b genotype and increased risk of CRA in the recessive model, particularly after adjustment for confounders, suggesting that this genotype may be a risk factor for the development of CRA. The discrepancy between the meta-analyses that show that the *BsmI* polymorphism modifies CRC risk [15,16] but not CRA risk [18] suggests that the BsmI mutation might be more involved in the later stages of CRC development. However, our results indicate that the B allele, with its protective effect against CRA, might play a role in the earlier stages of CRC development.

Obesity is a recognized risk factor for T2DM, which in turn is associated with an increased risk for CRA and CRC. Elevated levels of insulin and IGF-1 contribute to the accelerated growth of colon cells, potentially leading to cancer development. This risk is further heightened in patients treated with diabetic medications such as sulfonylureas and insulin. Moreover, high levels of glycated hemoglobin have been found to predict worse clinical outcomes in CRC patients [20]. The *INSR* A-603G promoter SNP has been found to modify the risk of CRC (with a protective effect from the G allele) [21]. We did not find an association between rs2059806 and the risk of CRA in our patients despite its association with T2DM [13].

Concerning the molecular mechanisms underpinning the link between vitamin D and insulin resistance, it has been demonstrated that vitamin D enhances insulin receptor expression in muscle, liver, and adipose tissue, thereby improving insulin sensitivity. Specifically, vitamin D functions as an epigenetic regulator, modulating the transcription of several genes critical to insulin sensitivity. For instance, in high-fat diet mouse models treated with vitamin D, there is a 2.4-fold increase in the expression of insulin receptor substrate, a key protein that amplifies insulin signaling. This upregulation of insulin receptor substrate enhances insulin sensitivity in target tissues. Furthermore, vitamin D augments the responsiveness of insulin receptors to insulin, facilitates glucose transport, and promotes the conversion of proinsulin to insulin. These molecular mechanisms collectively underscore the pivotal role of vitamin D in mitigating insulin resistance [22]. 

In our study, patients diagnosed with CRA were older than the controls. The incidence of CRA is significantly greater in patients older than 50 years, and the incidence increases with each decade of life after age [23]. In the United States, 11% of CRC cases in men are diagnosed in individuals under 50 years old, compared to 10% in women. In recent decades, there has been a consistent increase in the incidence of CRC, particularly rectal cancer, among younger patients. Projections suggest that by 2030, 10% of colon cancers and 22% of rectal cancers in the U.S. will be identified in patients younger than 50 years of age. The American Cancer Society recently recommended lowering the starting age for CRC screening from 50 to 45 years [24]. Similar to CRC, adenomas are more frequently found in men than in women, with studies often showing that the prevalence in men is nearly double that in women [25]. The pattern for serrated polyps according to age and sex is less clear. Some research suggests only a modest increase in prevalence with age, and findings indicate that sessile serrated polyps tend to be more common in women than in men. Furthermore, the location of adenomas within the colon varies by sex, with adenomas more likely to be found in the proximal colon in women [26]. In our study, women did not present with more frequent serrated adenomas and did not have a greater incidence of right colon CRA. The significant age differences observed in CRA patients with b/b and G/G genotypes suggest that these genetic markers may be associated with earlier disease onset. However, the lack of significant differences in the BMI, abdominal circumference, and specific adenoma characteristics indicates that for our study, these genotypes may not influence disease severity or manifestation beyond the age of onset. The observation that G/G genotype carriers who develop adenomas are younger should be viewed cautiously in the absence of an associated risk increase. The fact that adenomas in these patients present at a younger age might have implications for earlier surveillance if this finding is supported by further research.

Studies have established a correlation between obesity and CRC. An increased BMI poses a greater relative risk for CRC in men than in women. Additionally, abdominal obesity has a stronger association with CRC than fat accumulation in other body areas. Research also indicates that obesity onset at an early age is associated with increased CRC incidence [21]. An increase in the BMI classified as either overweight or obese is also linked to a greater frequency of CRA. Among these, adenomatous polyps with high-grade dysplasia, which closely resemble malignant conditions, have an increased incidence in overweight or obese patients [27]. Weight loss in adulthood is associated with a reduced risk of CRA, particularly in overweight or obese patients [28]. In our study, the lack of a difference between cases and controls for the BMI, as well as for abdominal circumference, did not allow us to measure their influence on CRA incidence in our population.

Tobacco, which contains carcinogens, is believed to cause permanent genetic harm to the colorectal mucosa, leading to the development of colorectal polyps. The link between cigarette smoking and the risk of developing polyps is significant across sex, polyp type, polyp location, and polyp severity. Smokers have a 2–3 times greater risk of polyps than nonsmokers. Evidence suggests a stronger association with sessile serrated polyps than with adenomatous polyps [29]. The case and control subjects in our study had a low prevalence of smoking, which did not allow confirmation of the previously mentioned results.

Limitations of this study include the small sample size. The study population consisted of individuals of Romanian nationality, which may limit the applicability of the findings to other populations with different genetic backgrounds, environmental exposures, or lifestyle factors. Conducting the study in a single hospital may introduce selection bias, as the patient population may not be representative of the broader community or other regions. The measurement of serum vitamin D levels would have provided further insight into the interaction between CRA incidence, vitamin D status, and VDR polymorphism. Future research should focus on investigating the association between polymorphisms and the stages of cancer progression. This includes exploring how VDR polymorphisms, such as BsmI, and INSR polymorphisms, such as NsiI A/G, influence the transition from colorectal adenoma to carcinoma. Longitudinal studies and large-scale, multi-population research are essential to understanding these genetic interactions and their implications for personalized medicine. The strength of our study lies in its investigation of the VDR-BsmI and NsiI A/G-INSR polymorphisms within a Romanian population. Consistent with findings from previous research, our results confirm that the b/b genotype of VDR-BsmI is a significant risk factor for CRA. Additionally, our study is the first to explore the association between the NsiI A/G-INSR polymorphism and CRA risk. Although the G/G genotype does not alter the risk of developing CRA, the fact that adenomas in these patients present at a younger age might provide guidance for further management.

## 4. Materials and Methods

### 4.1. Study Population

This case–control study included 110 individuals, comprising 67 subjects diagnosed with CRA and 43 control subjects. All participants were patients who underwent colonoscopic examination at University Hospital C.F.R. in Cluj-Napoca, Romania, from August 2020 to December 2021. The inclusion criterion for the case group was a histopathologically confirmed diagnosis of CRA following colonoscopy in adults aged over 30 years. Conversely, the control group included patients for whom colonoscopy ruled out the presence of CRA. The exclusion criteria for both patients and controls included a confirmed diagnosis of CRC or any other malignancy; a history of familial adenomatous polyposis, Gardner syndrome, Lynch syndrome, or Peutz–Jeghers syndrome either personally or in the family; a diagnosis of inflammatory bowel disease; any previous intestinal resection; supplementation with calcium or vitamin D prior to the study; and a diagnosis of T2DM. Additionally, information regarding smoking history was collected. The colorectal adenomatous polyps identified were classified into different pathological types: tubular adenoma, villous adenoma, tubulovillous adenoma, hyperplastic, and serrated. Histopathological diagnoses were confirmed at the same hospital.

### 4.2. DNA Isolation

Genomic DNA was extracted from 2 mL of blood drawn in tubes with EDTA using a Zymoresearch kit (Quick-DNAMiniprep, Kit-Zymo Research Corporation, Freiburg, Germany). The samples were stored at -20 °C until PCR preparation.

### 4.3. PCR-RFLP Reaction

Polymerase chain reaction (PCR) was used to determine the genotypes and allele frequencies of the VDR-BsmI and NsiI A/G-INSR polymorphisms. We used the methods described by Khalid et al. (2016) and Bagheri et al. (2014) [30,31].

To identify VDR-BsmI, PCR was performed in a 25 μL mixture containing 20 ng of genomic DNA, 0.2 μM forward and reverse primers, 2.0 mM MgCl2, 200 mM dNTPs, and 0.625 U of Taq polymerase. The sequences of the primers used were as follows: forward primer, 5′-CAA CCA AGA CTA CAA GTA CCG CGT CAG TGA-3′; reverse primer, 5′-AAC CAG CGG AAG AGG TCA AGG G-3′.

For NsiI A/G-INSR polymorphism identification, PCR was performed in a 25 μL mixture containing 20 ng of genomic DNA, 0.3 μM forward and reverse primers, 2.5 mM MgCl2, 200 mM dNTPs, and 0.625 U of Taq polymerase. The sequences of the primers used were forward primer 5′-CGG TCT TGT AAG GGT AAC TG-3′ and reverse primer 5′-GAA TTC ACA TTC CCA AGA CA-3′.

Primers were obtained from Eurogentec (Kaneka Eurogentec S.A. Biologics Division, Liege, Belgium). Amplification was performed in an iCycler C1000 Bio-Rad (Bio-Rad Life Science, Hercules, CA, USA) following a specific program for VDR-BsmI polymorphism identification: initial denaturation for 60 s at 95 °C, 34 cycles of denaturation for 10 s at 95 °C, primer annealing for 50 s at 67.9 °C and a final extension for 30 s at 72 °C. The amplified fragment was 825 bp in length. For NsiI A/G-INSR polymorphism identification, the following program was used: initial denaturation for 10 min at 95 °C, 34 cycles of denaturation for 10 s at 95 °C, annealing for 20 s at 53.4 °C, extension of the primers at 72 °C for 20 s and a final extension for 5 min at 72 °C. The amplified fragment was 324 bp in length. The specificity of the PCR amplification was checked by migration of the probes on a 2% agarose gel stained with 10 mg/l ethidium bromide solution.

**Figure 1:**
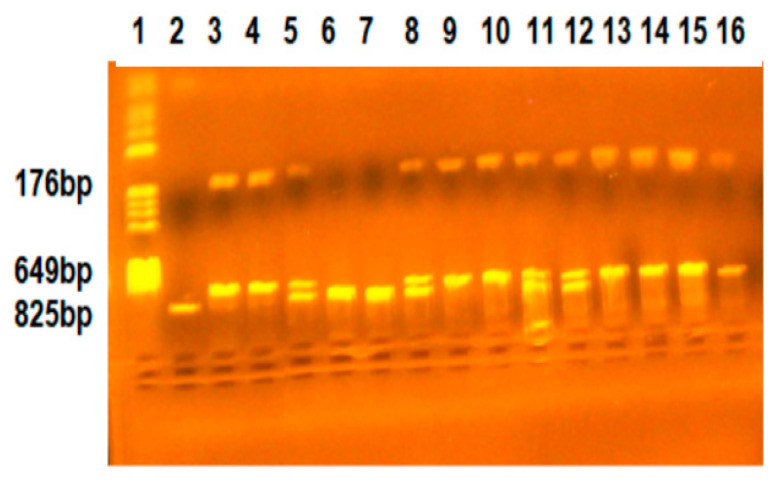
Identification of VDR-BsmI genotypes. Lane 1-pBRHaeIII-Digest DNA molecular marker; Lanes 2, 6, 7-BB genotype (fragment of 825 bp); Lanes 3, 4, 9, 10, 13, 14, 15, 16-bb genotype (fragments of 649 bp, 176 bp; Lanes 5, 8, 11, 12-Bb genotype (fragments of 825 bp, 649 bp, 176 bp).

For restriction fragment length polymorphism (RFLP) analysis, 6 μL of PCR products were digested with 2 U of BsmI and NsiI restriction endonucleases in a 10 μL mixture for 3 h at 37 °C. The BsmI polymorphism revealed two alleles, allele B (absence of a restriction site) and allele b (presence of a restriction site). The three genotypes were as follows: B/B (fragments of 825 bp), B/b (fragments of 825 bp, 649 bp, 176 bp), and b/b (fragments of 649 bp, 176 bp) (Figure 1). 

**Figure 2:**
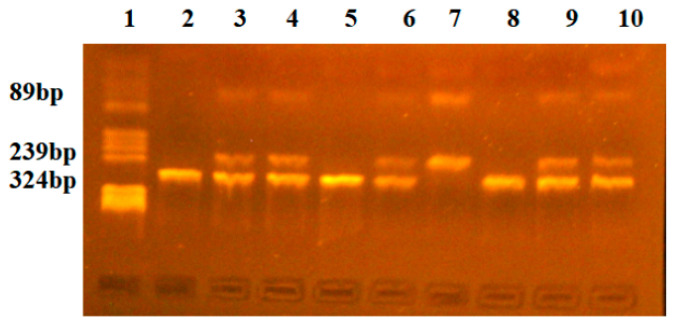
Identification of INSR-NsiI genotypes. Lane 1-pBRHaeIII-Digest DNA molecular marker; Lanes 2, 5, 8-GG genotype (fragment of 324 bp); Lanes 3, 4, 6, 9, 10-GA genotype (fragments of 324 bp, 239 bp; Lane 7-AA genotype (fragments of 239 bp, 89 bp).

The NsiI polymorphism revealed two alleles, allele G (absence of a restriction site) and allele A (presence of a restriction site). The three genotypes were as follows: G/G (fragments of 324 bp), G/A (fragments of 324 bp, 239 bp, and 89 bp), and A/A (fragments of 239 bp and 89 bp) (Figure 2).

The BsmI and NsiI restriction endonucleases were obtained from New England Biolabs (New England Biolabs UK, Ltd., Hitchin, UK).

### 4.4. Statistics

Statistical analyses were performed using GraphPad Prism software version 9.0.0 (121). Quantitative continuous variables that adhered to a Gaussian distribution were summarized using the arithmetic mean and standard deviation (SD). For quantitative continuous variables that deviated from a Gaussian distribution, the median value and the interquartile range (IQR) (Q1 represents the 25th percentile, and Q3 represents the 75th percentile) were used. Qualitative nominal variables were described using both relative percentages and absolute frequencies (number of cases). Comparisons of the distributions of demographic, lifestyle, and clinical variables

In relation to CRAs or variant genotypes, Student’s t test for independent samples, the Mann–Whitney U test, or Fisher’s exact test was used.

Genetic models (codominant, dominant, recessive, and overdominant) were constructed with SNPStats software (https://www.snpstats.net/ (accessed on 11 March 2024)) [32]. Models adjusted for age, sex, body mass index (BMI), smoking history, and adjusted odds ratios (ORs) with 95% confidence intervals (CIs) were also constructed. Differences in outcomes were considered to be statistically significant at *p* < 0.05. To adjust for multiple testing, *p* values were corrected using the false discovery rate method, and an estimated Q value was also calculated [33]. The Hardy–Weinberg equilibrium was tested for each polymorphism using the χ^2^ test.

## 5. Conclusions

In summary, this study identifies the b allele of the VDR-BsmI gene as a significant risk factor for colorectal adenoma (CRA) in the Romanian population, highlighting its association with earlier diagnosis. The findings also suggest that the G/G genotype of the NsiI A/G-INSR polymorphism, while not directly increasing CRA risk, is linked to earlier onset of the disease. The absence of a significant correlation between the INSR rs2059806 polymorphism and CRA suggests a limited role of this gene variant in colorectal adenoma susceptibility. These results emphasize the importance of genetic screening for VDR-BsmI polymorphisms in assessing CRA risk and potentially guiding earlier surveillance and preventive strategies in individuals with the b/b genotype. Future research should explore the mechanisms underlying these associations and consider larger, more diverse populations to validate these findings and further understand the interplay between genetic factors and colorectal adenoma development.

## Figures and Tables

**Table 1:** Distribution of demographic, clinical, and lifestyle characteristics in cases and controls.

	Male			Female			Male vs. Female	Case vs. Control	
	Case (41)	Control (21)	*p*	Case (26)	Control (22)	*p*	*p* Cases	*p* Control	*p*
Age (years) ^(a)^	66.8 ± 9.7	61.0 ± 10.4	0.03 *	64.2 ± 11.0	59.6 ± 9.7	0.13	0.3	0.6	0.006 *
BMI ^1^ (kg/m^2^) ^(a)(b)^	29.4 ± 3.9	28.3 ± 4.2	0.3	28.4 ± 5.6	29.1 (27.7–32.1)	0.18	0.3	0.18	0.73
Abdominal circumference (cm) ^(a)^	106.5 ± 12.6	102 ± 14.2	0.2	97.8 ± 12.6	101.3 ± 11.5	0.3	0.2	0.84	0.5
Smoking history	14 (34%)	6 (28%)	0.77	4 (15%)	2 (9%)	0.6	0.15	0.13	0.07
Multiple adenomas	13 (31%)			6 (26%)			0.58		
High-grade dysplasia	14 (34%)			6 (26%)			0.28		
Tubular adenoma	33 (80%)			18 (69%)			0.38		
Tubulovillous adenoma	4 (9%)			2 (7%)			0.99		
Villous adenoma	3 (7%)			1 (3%)			0.99		
Serrated adenoma	3 (7%)			3 (11%)			0.67		
Hyperplastic polyp	3 (7%)			6 (23%)			0.07		
Family history of CRC ^2^	3 (7%)	1 (4%)		2 (7%)	3 (13%)		0.99	0.6	0.73
Polyp > 1 cm	23 (56%)			13 (50%)			0.8		
Right colon polyp	14 (34%)			8 (30%)			0.99		

^1^ BMI–body mass index; ^2^ CRC–colorectal cancer, ^(a)^ mean ± standard deviation, ^(b)^ median (Q1, Q3), *-indicates a significant result with *p* value < 0.05.

**Table 2:** Allelic and genotypic distributions of SNPs of *VDR-BsmI* and *NsiI A/G-INSR* gene and association with risk of CRA1.

	Model	Genotype	Controls ^(a)^	Cases ^(a)^	OR (95% CI)	*p*	OR ^(b)^ (95% CI)	*p* ^(b)^
VDR-*BsmI*	Codominant	B/B	15 (34.9%)	14 (20.9%)	1.00	0.11	1.00	0.086
		B/b	20 (46.5%)	30 (44.8%)	1.61 (0.64–4.04)		1.37 (0.50–3.75)	
		b/b	8 (18.6%)	23 (34.3%)	3.08 (1.04–9.12)		3.43 (1.07–11.01)	
	Dominant	B/B	15 (34.9%)	14 (20.9%)	1.00	0.11	1.00	0.16
		B/b + b/b	28 (65.1%)	53 (79.1%)	2.03 (0.86–4.79)		1.94 (0.77–4.88)	
	Recessive	B/B + B/b	35 (81.4%)	44 (65.7%)	1.00	0.06	1.00	0.033 (Q-value 0.08) ^(c)^
		b/b	8 (18.6%)	23 (34.3%)	2.29 (0.91–5.73)		2.84 (1.04–7.72)	
	Overdominant	B/B + b/b	23 (53.5%)	37 (55.2%)	1.00	0.86	1.00	0.52
		B/b	20 (46.5%)	30 (44.8%)	0.93 (0.43–2.01)			
NsiI A/G-INSR	Codominant	A/A	5 (11.6%)	5 (7.5%)	1.00	0.5	1.00	0.6
		A/G	13 (30.2%)	27 (40.3%)	2.08 (0.51–8.47)		2.12 (0.48–9.37)	
		G/G	25 (58.1%)	35 (52.2%)	1.40 (0.37–5.36)		1.60 (0.40–6.50)	
	Dominant	A/A	5 (11.6%)	5 (7.5%)	1.00	0.54	1.00	0.41
		A/G + G/G	38 (88.4%)	62 (92.5%)	1.63 (0.44–6.01)		1.77 (0.45–6.93)	
	Recessive	A/A + A/G	18 (41.9%)	32 (47.8%)	1.00	0.46	1.00	0.82
		G/G	25 (58.1%)	35 (52.2%)	0.79 (0.36–1.71)		0.91 (0.39–2.08)	
	Overdominant	A/A + G/G	30 (69.8%)	40 (59.7%)	1.00	0.28	1.00	0.44
		A/G	13 (30.2%)	27 (40.3%)	1.56 (0.69–3.51)		1.41 (0.58–3.42)	

^(a)^-absolute number and percentage of individuals, ^(b)^–ORs and *p* values adjusted for age, sex and smoking status; ^(c)^ Q-value < 0.10 using the False Discovery Rate adjustment for multiple testing.

**Table 3:** Clinical comparisons between genotypes of *VDR-BsmI* and *NsiI A/G-INSR* in CRA ^1^ patients and controls.

Patients and Controls	VDR-*BsmI*			*NsiI A/G*-INSR		
	BB + Bb (n = 79)	bb (n = 31)	*p*	AA + AG (n = 50)	GG (n = 60)	*p*
Age (years) ^(a)^	64.63 ± 10.93	61.29 ± 8.97	0.13	65.66 ± 9.16	62.05 ± 11.25	0.07
BMI ^2^ (kg/m^2^) ^(a)^	29.6 ± 4.4	28.08 ± 4.99	0.12	28.74 ± 4.04	29.53 ± 5.03	0.49
Abdominal circumference (cm) ^(a)^	103.5 ± 12.54	100.1 ± 14.13	0.21	102.1 ± 12.46	103 ± 13.59	0.74
Smoking history ^(b)^	16 (24%)	7 (22%)	0.79	8 (16%)	15 (25%)	0.34
Family history of CRC ^3(b)^	7 (8%)	2 (6%)	0.99	4 (8%)	5 (10%)	0.99
**Subjects with CRA only**	**VDR-*BsmI***			***NsiI A/G*-INSR**		
	**BB + Bb (n = 44)**	**bb (n = 23)**	***p***	**AA + AG (n = 32)**	**GG (n = 35)**	***p***
Age (years) ^(a)^	67.66 ± 10.72	62.39 ± 8.59	0.04 *	69.16 ± 7.46	62.83 ± 11.62	0.01 *
BMI ^2^ (kg/m^2^) ^(a)^	29.5 ± 4.26	28.19 ± 5.31	0.27	28.12 ± 4.22	29.9 ± 4.91	0.11
Abdominal circumference (cm) ^(a)^	104.7 ± 12.71	100.2 ± 14.06	0.19	102.1 ± 13.91	104.1 ± 12.76	0.54
Smoking history ^(b)^	3 (6%)	2 (8%)	0.99	2 (6%)	3 (8%)	0.99
Family history of CRC ^(b)^	3 (6%)	2 (8%)	0.75	2 (6%)	3 (8%)	0.99
Multiple adenomas ^(b)^	15 (34%)	4 (17%)	0.25	11 (34%)	8 (22%)	0.41
High-grade dysplasia ^(b)^	16 (36%)	4 (17%)	0.16	9 (28%)	12 (34%)	0.43
Tubular adenoma ^(b)^	31 (70%)	20 (86%)	0.22	22 (68%)	29 (82%)	0.25
Tubulovillous adenoma ^(b)^	5 (11%)	1 (4%)	0.65	2 (6%)	4 (11%)	0.67
Villous adenoma ^(b)^	4 (9%)	0	0.29	3 (9%)	1 (2%)	0.34
Serrated adenoma ^(b)^	6 (13%)	1 (4%)	0.4	3 (9%)	4 (11%)	0.99
Hyperplastic polyp ^(b)^	6 (13%)	3 (13%)	0.99	6 (18%)	3 (8%)	0.29
Family history of CRC ^(b)^	7 (15%)	2 (8%)	0.99	4 (12.5%)	5 (14%)	0.99
Polyp > 1 cm ^(b)^	23 (52%)	13 (56%)	0.8	17 (53%)	19 (54%)	0.99
Right colon polyp ^(b)^	16 (36%)	6 (26%)	0.42	10 (31%)	12 (34%)	0.99

^1^ CRA–colorectal adenoma, ^2^ BMI–body mass index, ^3^ CRC–colorectal cancer, ^(a)^ mean ± standard deviation, ^(b)^-absolute number and percentage of individuals *-indicates a significant result with *p* value < 0.05.

## Data Availability

The raw data used in this study can be obtained upon reasonable request from Lucia M. Procopciuc (l.procopciuc@umfcluj.ro) and George Ciulei (george.ciulei@umfcluj.ro).
